# Perceived psychosocial health and its sociodemographic correlates in times of the COVID-19 pandemic: a community-based online study in China

**DOI:** 10.1186/s40249-020-00770-8

**Published:** 2020-10-26

**Authors:** Gan-Yi Wang, Shang-Feng Tang

**Affiliations:** 1 Henan Medical Products Administration & Center for ADR Monitoring of Henan, 127 Huayuan Road, Zhengzhou, 450008 Henan China; 2 School of Medicine and Health Management, Tongji Medical College, Huazhong University of Science and Technology, 13 Hangkong Road, Wuhan, 430030 Hubei China; 3 Center for Health Service Research in Rural Areas, Key Research Institute of Humanities & Social Sciences at Universities in Hubei Province, 13 Hangkong Road, Wuhan, 430030 Hubei China

**Keywords:** COVID-19, China, Mental health, Psychosocial stress

## Abstract

**Background:**

Coronavirus disease 2019 (COVID-19) pandemic has been affecting people's psychosocial health and well-being through various complex pathways. The present study aims to investigate the perceived psychosocial health and its sociodemographic correlates among Chinese community-dwelling residents.

**Methods:**

This cross-sectional survey was carried out online and using a structured questionnaire during April 2020. In total, 4788 men and women with the age range of 11–98 years from eight provinces in eastern, central and western China were included in the analysis. We adopted a tactical approach to capture three key domains of perceived psychosocial health that are more likely to occur during a pandemic including hopelessness, loneliness, and depression. Multiple regression method, binary logistic regression model and variance inflation factor (VIF) were used to conduct data analysis.

**Results:**

Respectively 34.8%, 32.5% and 44.8% of the participants expressed feeling more hopeless, lonely, and depressed during the pandemic. The percentage of all three indicators was comparatively higher among women than among men: hopelessness (50.7% vs 49.3%), loneliness (52.4% vs 47.6%), and depression (56.2% vs 43.8%). Being married was associated with lower odds of loneliness among men (odds ratio [*OR*] = 0.63, 95% *CI*: 0.45–0.90). Loneliness was negatively associated with smoking (*OR* = 0.67, 95% *CI*: 0.45–0.99) and positively associated with drinking (*OR* = 1.45, 95% *CI*: 1.04–2.02). Compared with those in the lowest income bracket (< CNY 10 000), men (*OR* = 0.34, 95% *CI*: 0.21–0.55) and women (*OR* = 0.36, 95% *CI*: 0.23–0.56) in the highest level of annually housed income (> CNY 40 000) had the lowest odds of reporting perceived hopelessness (*OR* = 0.35, 95% *CI*: 0.25–0.48). Smoking also showed negative association with depression only among men (*OR* = 0.63, 95% *CI*: 0.43–0.91).

**Conclusions:**

More than one-third of the participants reported worsening in the experience of hopelessness and loneliness, with more than two-fifth of worsening depression during the pandemic compared with before the outbreak. Several socioeconomic and lifestyle factors were found to be associated with the outcome variables, most notably participants' marital status, household income, smoking, alcohol drinking, existing chronic conditions. These findings may be of significance to treat patients and help them recover from the pandemic.

## Background

The World Health Organization declared the coronavirus disease 2019 (COVID-19) outbreak as a public health emergency of international concern (PHEIC) on January 30, 2020 [1]. Countries around the world have soon responded to the emergency through the adoption of various strategies to contain the outbreak such as cessation of local and international travels, foreclosure of non-essential businesses, home quarantine for at-risk population, and strict physical distancing [2]. The drastic changes in social and personal aspects of daily living are resulting in considerable degrees of psychosocial distress [3–5]. Based on the relevant review, the probability of related psychological problems has been significantly increased due to the uncertainty and fear associated with the epidemic, as well as the large-scale blockade and economic recession. In this context, even with the world's most advanced health care system, there are inherent difficulties in providing such a wide range of psychological care [6].

As China was one of the worst-hit areas, a series of assessments on the psychological state of residents were conducted in the early stage of the epidemic. The country has been struggling to meet the mental healthcare needs of the population. Although the country has been able to achieve considerable progress in terms of promoting its mental healthcare infrastructure and service delivery system, evidence suggests that the prevalence of population with psychological conditions has been increasing. Unhealthy lifestyle behavior, sociocultural environment, and demographic structure are the commonly cited factors that are fueling the mental health crisis. Notably, the necessity for maintaining constant physical distancing has most certainly deepened social isolation and inadequate community adhesion in a society where loneliness is already a grave concern among mental healthcare providers. Even among people who having the capable of maintaining adequate networking and having a healthy social life are being forced to self-isolate themselves from their beloved ones driven by the fear of cross-transmission of the virus. This is especially for frontline workers such as those involved in the healthcare and retail industry. China is among the most highly urbanized countries in Asia, with nearly two-thirds of the population residing in the ever-expanding cities. The country has shown a strong resolution to fight urban poverty so far. However, pockets of poverty-ridden communities are still common who are now being the hardest-hit by the economic repercussions of the pandemic [7–9]. A great majority of the urban population are directly employed in the labor-intensive industries, and they are at risk of falling into poverty.

The compounding effect of loss of income and the adverse health outcomes can be identified as a key contributor to the perceived psychosocial situation of the population. For low-income earning individuals and families, loss of income can translate to catastrophic expenditures even when it comes to affording basic commodities. Several researches have been published so far illustrating the psychosocial health consequences of economic poverty among children and the general population in China [10, 11]. In addition to the financial impact, the prolonged pandemic is affecting psychosocial well-being, for example, disruption in routine lifestyle, alterations in the environmental factors that are driving unhealthy behaviors such as less physical activity, higher scopes for smoking and drinking, inadequate supply of fresh and nutritious food, and longer screen-time and more addictive social media use among the younger population. They have been shown to be the negative association associated with mental health outcomes [12–18]. The elderly population need higher dependency and physical and emotional care, especially for those with chronic health conditions, lack of psychologically supportive environment and caregiving can lead to feelings of hopelessness and loneliness [19–22]. China's growing segment of the elderly population and the capacity of the healthcare system to meet their special physical and mental healthcare needs are rising concerns among health policymakers and researchers. Since the outbreak of the pandemic, several research studies have been published regarding the mental health issues among the Chinese population [19, 23–25]. However, the findings are still mixed, and the use of different domains of mental health and their measurement techniques make their generalization and contextual interpretation challenging for scholars. In this study, we adopted a tactical approach to capture three core domains of mental health that are more likely to occur during the pandemic and relate them to several proximal and distal factors to understand the relative contrasting contribution to each of the three constructs.

## Methods

### Study settings and sampling methods

In this study, residents in eastern, central, and western China were selected through directional stratification and convenience sampling. According to the epidemic prevalence of COVID-19 on April 1, 2020, the top two provinces and lower one province based on the number of cases were selected from each region. Therefore, Hubei, Hunan, and Shanxi provinces were selected from Central China, and Guangdong, Zhejiang, and Fujian provinces were selected from eastern China. Due to the similarities in local conditions and customs between Sichuan and Chongqing, only one of those two provinces were chosen with a comparatively higher prevalence in western China. According to the comprehensive influence of the city in each province, the provincial capital and another city were selected in each province. Sixty households from both rural and urban households in one city and all households aged over ten years were invited to participate in the online survey. A total of 7118 residents from 1920 households in 8 provinces (16 cities) were surveyed. Due to the low response of residents in Guangdong and Zhejiang provinces, only half of the households attended the survey.

Data collection was conducted from April 4 to April 15 of 2020, a project manager in each province was recruited to coordinate provincial survey training. Six local investigators were recruited according to their annual household income in each local city to send online questionnaires and control the quality of investigation process. Half of them were from urban areas and most were college students. After receiving training in online data collection, each investigator was asked to send online questionnaires to 20 local families on their social network, including friends, relatives, native classmates, and so on. Each eligible family member was invited to fill out an online questionnaire (powered by www.wjx.cn) on an average of 15 min. A secret gift was sent to encourage the participants to complete the submission through the WeChat 7.0.12 (Tencent computer system Co. Ltd. Shenzhen, China). Due to the limitations of objective factors such as age, education level, and space distance, residents may lose the ability to participate in the online survey. It was suggested to invite the young offspring living together to answer the questions according to their choice. If there was difficulty in investigating the surrounding 20 families, a supplementary survey was carried out by other investigator to complete the remaining household survey.

Meanwhile, the follow-up investigation of quality control measures was taken during the data collection process. (1) Conducted a preliminary survey, group, and trained the investigators. (2) Each researcher was independent, but the relationship between students at different learning stages were allowed in this investigation. (3) Before distributing the online questionnaire, the eligible family numbers of each household were used to generate a unique questionnaire number. (4) Questionnaires for each family were sent out. They investigators were asked to convey a message: "Those who carefully complete the questionnaire will receive a secret gift." Furthermore, many trap questions were set in the questionnaire to identify people who did not answer the questions carefully. (5) The project manager checked the quality of each questionnaire according to the threshold value of survey time exceeding 450 s and the consistency of the two groups of questions set in the questionnaire.

### Outcome and explanatory measurements

In this study, we adopted a tactical approach to capture three key domains of psychosocial health that are more likely to occur during a pandemic including hopelessness, loneliness, and depression.

The first outcome variable is perceived hopelessness, which is a commonly construct used in population-based studies as an indicator of psychosocial well-being such as depression and suicide [26, 27]. It has been studied in the context of predicts general health and social functioning among the population with mood disorders, showing the wider applicability of this construct in the context of psychological well-being. In this study, it was measured by the question: would you say since the beginning of the pandemic you have been feeling hopeless: same as before, little worse than before, far worse than before. Hopelessness is associated with increases in the risk of emotional maladjustment and a range of negative mood states, both in the general population and clinical settings [28].

The second outcome variable is perceived loneliness, which is measured by the question: would you say since the beginning of the pandemic you have been feeling lonely? Same as before, little worse than before, far worse than before. Loneliness is widely a prevalent phenomenon globally and has been a popular topic of research across various domains including chronic health conditions, psychological stress, and anxiety [29]. Loneliness is a common human emotion that is linked to feeling of insecurity, vulnerability, and isolation and is also associated with overall morbidity and mortality in adult populations. Although there is no universally agreed definition of loneliness, it is generally understood as not just being alone, but perceived feeling of lack of an attachment figure, social network, and absence of a circle of people that allows an individual to develop a sense of belonging, of company, of being part of a community [29, 30].

The third outcome variable is perceived depression which is measured by the question: would you say since the beginning of the pandemic you have been feeling depressed? Same as before, little worse than before, far worse than before.

A single-item measure of self-rated depression (SRD) is being used increasingly population-based health survey for its ease of application and high sensitivity to objectively measured health outcome including all-cause mortality among cognitively intact community-dwelling older adults [31]. One-item question for measuring general health condition is increasingly used in epidemiologic survey [1], and measure by questions like: "In general, would you say your mental health is: Excellent, Very Good, Good, Fair or Poor?" [32]

Explanatory variables included: age (11–20, 20–29, 30–39, 40–49, 50–59, 60–69, 70–79, 80 +); sex (male/female); marital status (not married/married); annual household income (< CNY 10 000, CNY 10 000–20 000, CNY 20 000–30 000, CNY 30 000–40 000, CNY > 40 000); occupation (white-collar/blue-collar/student and unemployed); smoker (no/yes); alcohol consumer (no/yes); has any chronic conditions (no/yes); residency (urban/rural); provinces/municipality (Hunan, Hubei, Shanxi, Chongqing, Gansu, Fujian, Zhejiang, Guangdong).

### Data analysis

Data analyses were performed using Stata version 16 (StataCorp, Texas, USA). The prevalence of the sample population reporting hopelessness, loneliness, and depression was presented as percentages. Following that, the relationship between the three outcome and explanatory variables was measured by multivariable regression methods. Given the dichotomous nature of the outcome variables, a binary logistic regression model was used to generate the odds ratios (*OR*) and their 95% confidence intervals (*CI*). The variance inflation factor (VIF) was used as a measure of collinearity to ensure that none of the predictor variables in the final model was highly associated with each other. All statistical tests were two-tailed and *P* values below 0.05 were considered statistically significant.

### Ethics statement

The protocol was reviewed and the ethical approval was obtained from the Ethics Committee of Tongji Medical College, Huazhong University of Science and Technology (2020S107). The oral informed consent was obtained from each participant before taking the online survey.

## Results

### Prevalence of perceived psychosocial health

A total of 6253 residents over the age of 10 years old completed the survey, of which 4788 were eligible. The participation ratio was 87.9% (6253/7118), and the valid participation ratio was 67.1% (4778/7118). Basic demographic characteristics and the prevalence of reporting hopelessness, loneliness, and depression were presented in Table 1. Respectively 34.8%, 32.5%, and 44.8% of the participants expressed feeling more hopeless, lonely, and depressed during the pandemic. The percentage of all three indicators was comparatively higher among women than among men: hopelessness (50.7% vs 49.3%), loneliness (52.4% vs 47.6%), and depression (56.2% vs 43.8%).

**Table 1:** Demographic and social capital related explanatory variables and the perceived psychosocial health

		Hopelessness				Loneliness				Depression			
	*n*	No		Yes		No		Yes		No		Yes	
		*n*	(%)	*n*	(%)	*n*	(%)	*n*	(%)	*n*	(%)	*n*	(%)
Age, years													
< 20	359	31	8.6	20	5.5	26	7.2	29	8.0	28	7.9	25	7.0
20–29	1464	416	28.4	506	34.6	403	27.5	541	37.0	397	27.2	509	34.8
30–39	504	46	9.0	67	13.3	50	9.9	59	11.8	41	8.2	67	13.4
40–49	910	179	19.7	161	17.7	193	21.2	132	14.5	187	20.5	156	17.1
50–59	699	109	15.6	89	12.7	109	15.6	88	12.6	114	16.4	87	12.4
60–69	388	32	8.2	31	7.9	32	8.1	31	8.0	32	8.1	31	8.1
70–79	351	28	7.9	22	6.2	28	8.1	20	5.8	30	8.7	20	5.7
80+	113	3	2.5	2	2.1	3	2.4	3	2.4	3	3.0	2	1.5
Sex													
Male	2248	1109	49.3	955	42.5	1049	46.7	1069	47.6	1113	49.5	984	43.8
Female	2540	1287	50.7	1461	57.5	1355	53.3	1332	52.4	1282	50.5	1428	56.2
Marital status													
Not married	1937	771	39.8	807	41.7	713	36.8	931	48.1	739	38.1	839	43.3
Married	2851	1716	60.2	1663	58.3	1802	63.2	1481	51.9	1764	61.9	1616	56.7
Living arrangement													
Alone	418	33	7.9	43	10.3	33	7.8	44	10.6	35	8.4	38	9.2
With family	437	403	92.1	392	89.7	403	92.2	391	89.4	400	91.6	397	90.8
Annual household income (CNY)													
< 10 000	2074	860	41.5	971	46.8	871	42.0	956	46.1	893	43.1	905	43.6
10 000–20 000	1735	642	37.0	603	34.8	630	36.3	627	36.1	625	36.0	634	36.5
20 000–30 000	579	72	12.4	66	11.5	74	12.8	62	10.7	73	12.6	67	11.5
30 000–40 000	193	8	4.0	8	4.1	8	4.0	8	4.2	7	3.7	9	4.4
> 40 000	207	11	5.1	6	2.9	10	5.0	6	3.0	10	4.7	8	3.9
Occupation													
White-collar	1317	370	28.1	347	26.4	371	28.2	345	26.2	372	28.3	350	26.6
Blue-collar	890	163	18.4	169	19.0	172	19.3	153	17.2	172	19.3	158	17.8
Student/unemployed	2581	1381	53.5	1410	54.6	1357	52.6	1463	56.7	1353	52.4	1438	55.7
Smoke													
No	3867	3118	80.6	3133	81.0	3152	81.5	3064	79.2	3114	80.5	2526	81.0
Yes	921	178	19.4	175	19.0	170	18.5	191	20.8	179	19.5	34	19.0
Alcohol													
No	339	240	70.9	240	70.7	241	71.2	237	70.0	239	70.6	171	71.0
Yes	1398	408	29.2	409	29.3	403	28.8	420	30.0	411	29.4	118	29.0
NCDs													
No	371	290	78.3	282	76.1	288	77.5	287	77.4	288	77.7	224	77.3
Yes	1078	234	21.8	258	24.0	242	22.5	244	22.6	241	22.3	53	22.7
Residency													
Urban	1723	623	36.1	615	35.7	606	35.2	650	37.7	630	36.6	220	35.3
Rural	3065	1958	63.9	1970	64.3	1988	64.9	1909	62.3	1944	63.4	1267	64.7
Province/municipality													
Hunan	791	139	17.5	116	14.7	141	17.9	108	13.7	144	18.3	20	14.4
Hubei	672	74	11.0	133	19.8	85	12.6	114	17.0	66	9.8	14	19.2
Shanxi	728	113	15.5	106	14.6	108	14.9	116	15.9	112	15.4	17	15.0
Chongqing	703	119	16.9	74	10.6	117	16.6	75	10.7	122	17.3	14	11.4
Gansu	334	26	7.9	18	5.3	25	7.4	20	6.0	27	8.0	2	5.8
Fujian	280	17	6.1	15	5.5	18	6.5	13	4.5	16	5.8	1	6.0
Zhejiang	587	76	12.9	65	11.0	73	12.4	70	12.0	75	12.8	9	11.6
Guangdong	693	85	12.3	129	18.6	81	11.7	140	20.2	88	12.7	14	16.7

*NCDs* non-communicable diseases

**Fig. 1:**
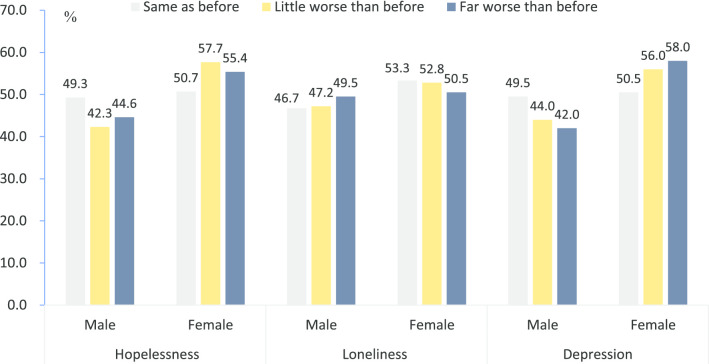
Prevalence of hopelessness, loneliness, and depression before and since the outbreak

As shown in Fig. 1, men were less likely to report same level of hopelessness (49.3% vs 50.7%), loneliness (46.7% vs 53.3%), and depression (49.5% vs 50.5%) during the pandemic than before compared with women. More than half of the women reported having a higher level of hopelessness (55.4%), loneliness (50.5%), and depression (58.0%) during the pandemic than before.

### The correlates of perceived psychosocial health

Factors associated with perceived psychosocial health were presented in Table 2. In general, compared with those in the youngest age group (11–19 years), those in the higher age groups had relatively higher odds of reporting hopelessness, loneliness, and depression. However, these associations reversed for those in the higher age groups of 70–79 and 80 + years old. For instance, participants aged 70–79 years had lower odds of reporting hopelessness (*OR* = 0.55, 95% *CI*: 0.37–0.82), loneliness (*OR* = 0.59, 95% *CI:* 0.39–0.87) and depression (*OR* = 0.53, 95% *CI:* 0.36–0.77). Women had higher odds of reporting all three outcomes, however, the odds were significant for depression only (*OR* = 1.38, 95% *CI:* 1.20–1.58). Those who were currently married had lower odds of loneliness (*OR* = 0.74, 95% *CI:* 0.59–0.92), with the association being significant among men only (*OR* = 0.63, 95% *CI:* 0.45–0.90). Household income showed consistently significant and inverse association with reporting hopelessness, but not with loneliness and depression. Compared with those in the lowest income bracket (< CNY 10 000), those in the highest (> CNY 40 000) had the lowest odds of reporting hopeless (*OR* = 0.35, 95% *CI:* 0.25–0.48), with the association being significant both among men (*OR* = 0.34, 95% *CI:* 0.21–0.55) and women (*OR* = 0.36, 95% *CI:* 0.23–0.56).

**Table 2:** Demographic and social capital related factors associated with hopelessness, loneliness, and depression

	Hopelessness *OR* 95% *CI*			Lonely *OR* 95% *CI*			Depression *OR* 95% *CI*		
	Pooled	Men	Women	Pooled	Men	Women	Pooled	Men	Women
Age, years (11–19)									
20–29	2.09*** 1.63–2.69	1.90** 1.29–2.80	2.26*** 1.62–3.15	1.10 0.86–1.42	1.06 0.72–1.56	1.14 0.81–1.60	1.35* 1.06–1.72	1.55* 1.05–2.27	1.28 0.93–1.77
30–39	3.24*** 2.26–4.65	3.95*** 2.27–6.87	2.84*** 1.75–4.62	1.25 0.87–1.78	1.16 0.68–1.98	1.39 0.86–2.26	1.87*** 1.33–2.65	2.17** 1.28–3.68	1.80* 1.13–2.88
40–49	2.23*** 1.57–3.16	2.73*** 1.57–4.75	2.00** 1.26–3.15	0.75 0.53–1.07	0.86 0.50–1.48	0.70 0.44–1.13	0.94 0.67–1.32	1.10 0.65–1.89	0.90 0.58–1.39
50–59	1.63** 1.14–2.33	2.03* 1.17–3.55	1.39 0.86–2.24	0.78 0.54–1.13	0.79 0.45–1.38	0.83 0.51–1.37	0.75 0.53–1.06	0.99 0.58–1.71	0.63 0.39–1.00
60–69	0.94 0.64–1.37	1.06 0.59–1.90	0.87 0.52–1.46	0.83 0.56–1.22	0.84 0.47–1.51	0.88 0.52–1.48	0.81 0.56–1.18	1.08 0.61–1.90	0.70 0.43–1.16
70–79	0.55** 0.37–0.82	0.64 0.36–1.16	0.51* 0.30–0.86	0.59** 0.39–0.87	0.67 0.37–1.21	0.54* 0.31–0.94	0.53*** 0.36–0.77	0.62 0.34–1.11	0.50** 0.30–0.84
80+	0.51** 0.31–0.84	0.44* 0.20–0.99	0.59 0.31–1.16	0.78 0.48–1.27	0.88 0.42–1.84	0.76 0.39–1.49	0.39*** 0.24–0.64	0.42* 0.19–0.93	0.40** 0.21–0.76
Sex (male)									
Female	1.15 1.0–1.33			1.06 0.91–1.22			1.38*** 1.20–1.58		
Currently married (no)									
Yes	1.18 0.94–1.47	1.07 0.74–1.53	1.26 0.94–1.67	0.74** 0.59–0.92	0.63* 0.45–0.90	0.78 0.59–1.05	0.98 0.79–1.21	0.91 0.65–1.29	1.00 0.76–1.32
Annual household income (< CNY 10 000)									
CNY 10 000–20 000	0.74*** 0.64–0.85	0.76* 0.61–0.95	0.72** 0.59–0.88	1.07 0.92–1.24	1.20 0.97–1.49	0.98 0.80–1.20	1.09 0.95–1.26	1.09 0.88–1.34	1.09 0.90–1.32
CNY 20 000–30 000	0.45*** 0.37–0.56	0.46*** 0.34–0.63	0.45*** 0.34–0.60	0.95 0.76–1.18	1.15 0.84–1.58	0.79 0.58–1.08	1.00 0.81–1.23	0.85 0.63–1.16	1.14 0.86–1.51
CNY 30 000–40 000	0.48*** 0.35–0.67	0.50** 0.32–0.80	0.46** 0.29–0.73	1.17 0.84–1.63	1.20 0.75–1.92	1.09 0.68–1.77	1.27 0.93–1.74	1.39 0.89–2.18	1.11 0.71–1.76
> CNY 40 000	0.35*** 0.25–0.48	0.34*** 0.21–0.55	0.36*** 0.23–0.56	0.70 0.49–1.01	0.72 0.43–1.21	0.72 0.44–1.18	0.99 0.72–1.35	1.05 0.67–1.66	0.96 0.62–1.48
Occupation (White-collar)									
Blue-collar	2.52*** 2.05–3.09	2.83*** 2.13–3.76	2.28*** 1.70–3.07	1.12 0.91–1.38	1.12 0.85–1.49	1.09 0.80–1.49	1.34** 1.11–1.63	1.36* 1.04–1.78	1.31 0.98–1.74
Student/Unemployed	1.38*** 1.17–1.64	1.44** 1.11–1.87	1.38** 1.10–1.74	1.09 0.92–1.30	1.03 0.79–1.34	1.09 0.86–1.39	1.38*** 1.17–1.63	1.28 1.00–1.65	1.49*** 1.19–1.86
Smoking (no)									
Yes	0.90 0.64–1.26	0.84 0.58–1.22	0.98 0.38–2.53	0.73 0.51–1.04	0.67* 0.45–0.99	0.90 0.34–2.37	0.71* 0.51– 0.99	0.63* 0.43–0.91	1.40 0.56–3.46
Drinking (no)									
Yes	1.18 0.85–1.66	1.29 0.87–1.91	0.90 0.45–1.78	1.45* 1.04–2.02	1.54* 1.05–2.28	1.22 0.62–2.43	1.47* 1.06–2.03	1.40 0.96–2.06	1.58 0.82–3.05
Has NCDs (no)									
Yes	1.07 0.89–1.27	1.04 0.81–1.33	1.09 0.84–1.42	1.33** 1.11–1.59	1.43** 1.11–1.84	1.24 0.95–1.62	1.52*** 1.28–1.80	1.53*** 1.21–1.95	1.49** 1.16–1.92
Residency (rural)									
Urban	1.57** 1.18–2.08	1.43* 1.05–1.97	2.29* 1.03–5.07	2.15* 1.17–3.96	1.80** 1.16–2.78	1.51 0.94–2.41	6.55* 1.24–34.74	1.16 0.87–1.55	1.39 0.69–2.80
Province (Hunan)									
Hubei	1.66*** 1.33–2.08	1.87*** 1.34–2.60	1.51** 1.11–2.05	1.75*** 1.40–2.20	1.63** 1.17–2.28	1.84*** 1.34–2.52	2.55*** 2.04–3.17	2.55*** 1.85–3.52	2.60*** 1.92–3.53
Shanxi	1.31* 1.05–1.63	1.44* 1.04–2.01	1.22 0.90–1.65	1.35** 1.08–1.70	1.35 0.96–1.88	1.33 0.97–1.83	1.28* 1.03–1.58	1.43* 1.04–1.97	1.17 0.87–1.57
Chongqing	0.85 0.68–1.06	0.89 0.64–1.23	0.81 0.60–1.10	0.86 0.68–1.10	0.89 0.62–1.26	0.84 0.60–1.17	0.86 0.69–1.08	0.93 0.67–1.29	0.80 0.60–1.08
Gansu	0.76 0.58–1.00	0.70 0.46–1.07	0.83 0.57–1.20	1.12 0.83–1.50	1.16 0.75–1.79	1.04 0.69–1.56	0.96 0.73–1.27	1.26 0.84–1.90	0.79 0.54–1.15
Fujian	1.03 0.77–1.39	1.22 0.77–1.92	0.92 0.63–1.35	0.89 0.65–1.23	1.09 0.67–1.76	0.74 0.48–1.15	1.33 –1.77	1.49 0.96–2.33	1.25 0.86–1.81
Zhejiang	0.93 0.74–1.17	0.84 0.60–1.19	1.03 0.75–1.40	1.28* –1.62	1.23 0.86–1.77	1.31 0.94–1.82	1.18 0.94–1.48	1.02 0.72–1.44	1.32 0.98–1.79
Guangdong	1.14 0.91–1.42	1.30 0.93–1.83	1.04 0.76–1.42	1.97*** 1.57–2.48	1.99*** 1.42–2.79	1.92*** 1.40–2.63	1.60*** 1.28–1.98	1.65** 1.19–2.28	1.53** 1.14–2.06
Total	4788	2248	2540	4788	2248	2540	4788	2248	2540

*OR* odds ratio, *CI* confidence interval

Level of significance: *P < 0.05, **P < 0.01, ***P < 0.001. The items in the brackets were the referred subgroups

Participants who were employed in blue-collar jobs, as well as those with no job or studying, had higher odds of reporting hopelessness and depression. In terms of hopelessness, the association with occupation was significant among men (*OR* = 1.36, 95% *CI*: 1.04–1.78). In terms of depression, the association with occupation was significant among women (*OR* = 1.49, 95% *CI*: 1.19–1.86). Smoking was negatively associated with loneliness (*OR* = 0.67, 95% *CI*: 0.45–0.99), but it was negatively associated with depression only among men (*OR* = 0.63, 95% *CI:* 0.43–0.91). However, the positive association with drinking was also found (*OR* = 1.45, 95% *CI:* 1.04–2.02).

Having NCDs was associated with higher odds of reporting loneliness among men (*OR* = 1.43, 95% *CI:* 1.11–1.84), and of reporting depression both among men (*OR* = 1.53, 95% *CI:* 1.21–1.95) and women (*OR* = 1.49, 95% *CI:* 1.16–1.92). Participants in the urban areas had higher odds of reporting hopelessness (*OR* = 1.57, 95% *CI:* 1.18–2.08), loneliness (*OR* = 2.15, 95% *CI:* 1.17–3.96) and depression (*OR* = 6.55, 95% *CI:* 1.24–34.74). Compared with Hunan province, Hubei province had higher odds of reporting all three outcomes both among men and women. While Guangdong province had higher odds of reporting loneliness among men and women.

## Discussion

More than one-third of the participants reported worsening in the experience of hopelessness and loneliness, with more than two-fifth of worsening depression during the pandemic compared with the time before. Notably, the percentage of the perceived hopelessness, loneliness, and depression was comparatively higher among women than among men, implying that gender-gradient in the vulnerability to mental health implications of the pandemic. There is a growing volume of literature on mental health repercussions of the pandemic, but the sex-differences in mental health-related outcomes are not very clear. However, the prevalence of psychological disorders, especially that of major depressive disorders has been found to be higher among women in previous study [33]. In the context of COVID-19, women might be at higher risk of poor mental health outcomes due to issues related to increased incidence of intimate partner violence (IPV) and loss of livelihood. Besides, women who are pregnant and experiencing difficulties in receiving routine antenatal care may experience psychological challenges that are being ignored by themselves and their caregivers. Unfortunately, these potential factors such as pregnancy, quality of marriage were not included in this study. Therefore, it recommends that future research should underscore these issues to better understand the sex-disparity in mental health outcomes from COVID-19.

This study also revealed that the exacerbation of experience of hopelessness, loneliness, and depression are correlated with a range of sociodemographic and economic factors. We found that participants in the higher age groups had relatively higher odds of reporting hopelessness, loneliness, and depression, except for those in the oldest age groups (70+ years), in whom the association was reversed. In general, this study show that being married, living in a high-income family, and working in a white-collar job all have protective effects on these three outcomes. Expectedly, we found a strong positive association between reporting hopelessness and household income. The current body of literature provided evidence of the physical and psychological morbidities resulting from financial constraints [34, 35], and a handful of studies briefly focused on the construct of hopelessness [36–38]. The intersection between financial and mental well-being is mediated with the underlying benefits of material advantage. Nonetheless, this result should be interpreted with caution since we had data only on raw income which may not be indicative of the actual financial situation of the participants. It was also worthy of noting that household income didn't show any significant association with loneliness and depression. While the link between socioeconomic status and mental health is relatively clear, our findings enrich the literature by showing contrastingly that annual housed income are more likely to be correlated with a sense of hopelessness.

Regarding health and health related behavior, we found that tobacco smoking was negatively associated with loneliness and depression, while drinking was positively associated with loneliness. Several studies have so far discussed that the use of both smoking and drinking are being triggered by the psychosocial stress resulting from the pandemic [17, 18, 39, 40]. Having NCDs was also found to be associated with higher odds of reporting loneliness and depression both among men and women. In China, NCDs represent a major contributor to mental health related morbidities and mortalities especially among the elderly population [41–43], and the current situation is likely to be further aggravating given the higher susceptibility of the elderly population to COVID-19 infection. While the healthcare system is being overstrained with COVID-19 patients, the mental healthcare needs of people with chronic diseases should be given special priority at the same time. Lastly, the participants in the urban areas had higher odds of perceived psychosocial health, indicating that the urban population share a higher susceptibility to psychological stressors compared with their rural counterparts. The underlying reasons behind this urban–rural difference might be rooted in factors such as population density and relative risk of cross-transmission, differences in the type of employment, and availability of essential goods and services.

In light of the above, it is important to provide the necessary mental health support [44]. We recommend the active and ongoing participation of mental health professionals in policy task forces during this critical period [45].To meet the needs of the general population during this pandemic, it is necessary to consider online or smartphone-based psychosocial education to promote mental health and psychological interventions [46, 47], such as cognitive behavioral therapy (CBT) and mindfulness-based cognitive therapy (MBCT). MBCT focuses on the use of various mindfulness meditation exercises to develop a sense of right and wrong judgment and is particularly helpful in relieving stress in people with poor physical conditions. In addition, online platforms are well suited to isolating people and can be a way for people to offer support to each other, sharing their challenges and solutions during an outbreak to ease their anxiety and depression [48].

### Strengths and limitations

The sample size of this cross-sectional survey was relatively large and included participants with a broad age range. One important aspect of the study is the contrasting measurement of the outcome factors before and during the pandemic. This method of subjective measures of mental health status is relatively simpler and yet captures important information regarding the change in the situation specific to the pandemic. It should be kept in mind that this method doesn't reflect whether or not people were in sound mental health status prior to the pandemic, but rather the shift which can be used effectively in other crisis settings such as natural disasters. Our results should be interpreted with caution because of several limitations. First, the data is cross-sectional and the associations cannot indicate causality. Second, the conclusions cannot be generalized to the entire population of China due to inadequate sample size. Third, data was self-reported, and therefore the chance of reporting bias cannot be ignored. We were also unable to include these potential factors such as pregnancy and spousal relationships which are likely to be associated with the outcome variables among women. Also, the financial situation was not measured as a subjective assessment of solvency, which could have given a better reflection of the association between material wealth and psychological health.

## Conclusion

Findings showed that more than one-third of the participants reported worsening in the experience of hopelessness and loneliness, with more than two-fifth of worsening depression during the pandemic compared with the time before, with the percentage of all three indicators being comparatively higher among women than among men. Several socioeconomic and lifestyle factors were found to be associated with the outcome variables, most notably participants' marital status, household income, smoking, alcohol drinking, existing chronic conditions. Although the data are cross-sectional and hence no causal inference can be made of the associations, our study makes an important contribution to the current literature regarding the mental health situation among the population who are not directly affected by the pandemic, but among the healthy and community-dwelling population. These findings will help to understand the sociodemographic groups sharing a higher susceptibility to psychosocial stress arising from the pandemic and design proper intervention strategies.

## Data Availability

The datasets used and/or analyzed during the current study are available from the corresponding author on reasonable request.
